# Case report: An unusual presentation of acute promyelocytic leukemia in a middle aged female mimicking dengue infection

**DOI:** 10.1097/MD.0000000000021011

**Published:** 2020-06-26

**Authors:** Laura Yapor, Maleeha Zahid, Nikee Shrestha, Randee Walck, Zwi Schreiber, Muhammad Adrish

**Affiliations:** a Department of Medicine, Bronx Care Health System, Affiliated with Icahn School of Medicine at Mount Sinai, 1650 Grand Concourse, Bronx, NY; b American University of the Caribbean, Jordan Dr, Sint Maarten, FL; c Division of Hematology, Department of Medicine, Bronx Care Health System, Affiliated With Icahn School of Medicine at Mount Sinai, 1650 Grand Concourse, Bronx, NY; d Division of Pulmonary and Critical Care Medicine, Department of Medicine, Bronx Care Health System, Affiliated With Icahn School of Medicine at Mount Sinai, 1650 Grand Concourse, Bronx, NY.

**Keywords:** acute myeloid leukemia, acute promyelocytic leukemia, case report, dengue virus infection

## Abstract

**Rationale:**

Acute promyelocytic leukemia (APL) is an uncommon subtype of acute myeloid leukemia (AML). M3v phenotype is a less common presentation of APL and these patients usually present with leukocytosis and abnormal promyelocytes that are characterized by sparse granulation and are less likely to have faggot cells with multiple Auer rods. Distinguishing M3v phenotype from acute febrile illness can be challenging as the diagnosis relies on examination of peripheral smear.

**Patient concerns:**

Fifty-seven-year-old female who presented after recent trip to Dominican Republic for high grade fever and gum bleeding. She was exposed to patients with Dengue fever during her stay. At presentation, patient had leukocytosis, thrombocytopenia, and urinalysis showing bacteria and white cell. She was started on treatment for urinary tract infection. Patient remained febrile and thrombocytopenia worsened. On day 2, flow cytometry of the peripheral smear showed 43% medium sized blasts. Fluorescence in situ hybridization was positive for promyelocytic leukemia/retinoic acid receptor alpha.

**Diagnoses:**

The patient was diagnosed with APL.

**Interventions:**

Patient was started on treatment with all-trans retinoic acid and arsenic trioxide along with supportive care

**Outcomes:**

Patient had a favorable clinical response and her symptoms subsided.

**Lessons:**

Flow cytometry of the peripheral smear is key to diagnosis of suspected APL. One must maintain high suspicion for this life-threatening condition as early diagnosis saves lives.

## 1 Introduction

Acute myeloid leukemia (AML) is a heterogenous malignancy which is characterized by impaired differentiation and clonal proliferation of myeloid precursor cells. Median age of the patients at the time of diagnosis ranges from 65 to 70 years.^[1]^ Acute Promyelocytic Leukemia (APL) accounts for 5% to 8% of cases of AML and is typically characterized by neoplastic proliferation of bone marrow precursor cells to promyelocytic phenotype.^[2]^ These patients frequently present with leukopenia and thrombocytopenia leading to bleeding complications. Here we present a case of middle-aged female preexisting thrombocytopenia due to liver cirrhosis who presented with fever and leukocytosis after exposure to Dengue virus.

## 2 Case presentation

Our patient was a 57-year-old female who presented for high grade fever and gum bleeding. Patient returned from her trip to Dominican Republic five days prior to presentation where she was reportedly exposed to sick contacts with Dengue infection. Her past medical history was significant for Hepatitis C, anemia, chronic thrombocytopenia due to hepatitis C, and recurrent renal calculi. No close family members with history of malignancy. Patient also reported fatigue, headache, diffuse body aches and hematuria. On examination patient had temperature 103°F, pulse rate of 115/min, and blood pressure was 147/90 mm Hg. Patient appeared pale with multiple ecchymotic bruises on her extremities. Her lungs were clear, and no organomegaly was noted on abdominal examination. Laboratory examination revealed hemoglobin of 12.4 g/dl, white cell count of 16.3 cubic millimeters of blood (k/ul), platelet count of 45 k/ul, international normalized ratio of 1.6 and partial thromboplastic time of 29.6 seconds. Biochemical profile revealed normal electrolytes, renal function, and liver function test. C-reactive protein was elevated to 9.83 mg/L. Urinalysis was positive for bacteria and leukocyte esterase and patient was started on IV ceftriaxone for suspected urinary tract infection. Patient continued to have daily high-grade fever with progressive thrombocytopenia along with coagulopathy and leukocytosis. Further workups for infectious etiology including HIV infection, malaria, dengue, leptospira, enteric fever came back negative. Connective tissue workup including ANA and rheumatoid factor was negative so was the hemoglobin electrophoresis. Patient's fibrinogen level was noted to be low at 85 mg/dl and patient was diagnosed with disseminated intravascular coagulation (DIC). Flow cytometry of peripheral blood was performed, which revealed moderate leukocytosis with 43% medium sized blasts. A predominant myeloblast population was seen which was positive for CD34, CD13, CD33 (Dim) and CD117 with a subset having aberrant CD56 expression. These findings were consistent with diagnosis of acute myeloid leukemia. Fluorescence in situ hybridization for promyelocytic leukemia/retinoic acid receptor alpha (PML-RARα) was positive in 96% of cells tested; consistent with the hypogranular variant of acute promyelocytic anemia. FISH for t (8, 21) was negative. Patient was transferred to hematology unit where she was Fluorescence in situ hybridization started on treatment with all-trans retinoic acid (ATRA) and arsenic trioxide (ATO) along with supportive care and had a favorable response.

## 3 Discussion

Dengue virus infection is a common mosquito-borne viral disease in the world today. Classical dengue fever is an acute infection which present 4 to 10 days after the bite of an infected mosquito.^[3,4]^ The initial febrile phase is characterized by rapid onset high-grade fever which can be accompanied by headache, generalized body ache, malaise, joint and muscle pain along with skin erythema and photophobia. The febrile phase can also have concurrent hemorrhagic symptoms that can range from a positive tourniquet test and petechiae to spontaneous bleeding from the mucosal surfaces. Our patient presented 5 days after known exposure to Dengue virus patients. Her presenting symptoms of high-grade fever, fatigue, headache, diffuse body aches, gum bleeding and hematuria mimicked dengue virus infection. However, we were able to quickly narrow down the differential and diagnose her with APL soon after admission.

APL was first described by a Norweigian hematologist Leif Hillsted in 1957 when he described three patients who had rapid fatal course. These patient's white cell count smears were dominated by promyelocytes and had bleeding tendency due to thrombocytopenia and fibrinolysis.^[5]^ Bernard described 20 more cases of APL in 1959 where the predominant malignant cells were promyelocytes with immature nucleus and azurophilic granules in the cytoplasm.^[6,7]^ These cells were formally categorized by French-American-British as M3 cells in 1976.^[8]^ Four years later, a microgranular variant (M3v) of promyelocytes with bilobed nuclei no visible granules on light microscopy was identified.^[9]^ Patients with M3 phenotype usually present with leukopenia, abnormal promyelocytes with abundant cytoplasmic granules and identifiable faggot/matchstick cells with numerous Auer rods and accounts for up to 70% of APL cases. M3v phenotype is a less common presentation of APL and these patients usually present with leukocytosis and abnormal promyelocytes that are characterized by sparse granulation and are less likely to have faggot cells with multiple Auer rods.^[2]^

A specific reciprocal translocation involving long arms of chromosomes 15 and 17 is seen with this condition. The translocation involves PML gene on chromosome 15 and retinoic acid receptor alpha (RARα) on chromosome 17 generating two fusion genes PML/RARα and the reciprocal RARα/PML gene. APL usually presents in younger population with median age around 40 to 45 years at diagnosis compared to AML where median age is around 70.^[10]^ Approximately 80% patients present with consumptive coagulopathy which can have wide ranging manifestation from mild petechiae, bruising to more severe internal bleeding.^[11]^ Mortality rates also differ when comparing real world situation (17%–29%) to those of patients in clinical trials (about 5%).^[12,13]^ This further emphasizes the early diagnosis and immediate institution of treatment that is critical for survival of these patients.

Role of anthracycline chemotherapy was established by studies in 1980s when APL cells were noted to be sensitive to daunorubicin and the favorable response of AML cells to cytarabine was noted.^[14,15]^ These chemotherapeutic regimens were able to obtain a cure rate of 80% in newly diagnosed patients and 5-year disease free survival of 35% to 45%.^[16]^ However, early death was reported in about 15% of the patients undergoing treatment with this regiment mainly attributed to aggravation of hemorrhagic diathesis. In 1986, Daenen et al administered 13-cis-retinoid acid to a patient with APL who could not get chemotherapy due to active aspergillus infection and signs of coagulopathy disappeared.^[17]^ Subsequent trials in China, France, and US confirmed cute rates of about 95% with ATRA therapy, however ATRA alone led to relapse in 3 to 6 months.^[18,19]^ Study by Fenaux et al compared the role of ATRA plus chemotherapy with ATRA followed by chemotherapy and noted that those who received ATRA plus chemotherapy had lower relapse rate.^[20]^ Subsequent large series showed 5-year event free survival of 70% with this treatment.

Differentiation syndrome (previously known as retinoic acid syndrome) is a common but serious complication of ATRA and/or ATO therapy and can occur within the first few days or weeks of treatment. Patients present with shortness of breath, fever, hypotension, weight gain, acute renal failure, pleural effusion, interstitial infiltrates, and peripheral edema.^[21]^ Most recent regimens include steroid prophylaxis for differentiation syndrome which is also the treatment. Temporary discontinuation of ATRA/ATO therapy is only indicated in severe cases.^[22]^ Other treatment related complications include pseudotumor cerebri, hyperleukocytosis, hepatotoxicity and QT prolongation. Leukapharesis is usually avoided in patients with hyperleukocytosis due to risk of precipitating fatal hemorrhage.^[23]^

Our case is unusual as this patient was relatively older compared to median age at presentation for APL and presented with the less common type of APL that is known to have leukocytosis rather than commonly seen leukopenia. She had a history of hepatitis C and thrombocytopenia due to which initial suspicion for APL was low. Patient had multiple renal stones in the past and in the presence of positive urinalysis, this raised the suspicion for sepsis due to urinary tract infection. Patient also reported history of recent exposure to Dengue with signs and symptoms mimicking Dengue virus infection.^[3]^ Most of these were present in our patient at presentation. Nonetheless, we were able to quickly diagnose our patient with APL and started on ATRA/ATO treatment.

## 4 Conclusion

We present an unusual case of APL in a middle-aged female with recent Dengue exposure and many other confounders at presentation. One must maintain high suspicion for this life-threatening condition as early diagnosis saves lives.

## Author contributions

**Conceptualization:** Laura Yapoor, Maleeha Zahid, Nikee Shrestha, Randee Walck, Zwi Schreiber.

**Data curation:** Laura Yapoor, Maleeha Zahid, Nikee Shrestha, Randee Walck.

**Writing – original draft:** Laura Yapoor, Maleeha Zahid, Nikee Shrestha, Randee Walck.

**Writing – review & editing:** Laura Yapoor, Maleeha Zahid, Nikee Shrestha, Randee Walck, Zwi Schreiber.
